# Acceptability and feasibility of the Zulfiqar Frailty Scale (ZFS) in primary care: a cross-sectional survey

**DOI:** 10.25122/jml-2025-0175

**Published:** 2026-03

**Authors:** Abrar-Ahmad Zulfiqar

**Affiliations:** 1 Internal Medicine Department, Hôpitaux Universitaires de Strasbourg, Strasbourg, France

**Keywords:** frailty, primary care, screening, feasibility, Zulfiqar Frailty Scale

## Abstract

Frailty in older adults is a major public health concern in primary care. Although numerous frailty screening tools have been developed, their implementation in routine outpatient practice remains limited, mainly due to time constraints and perceived complexity. The Zulfiqar Frailty Scale (ZFS) was specifically designed for use in primary care. Beyond its psychometric properties, its acceptability and feasibility among healthcare professionals require evaluation. We conducted a cross-sectional descriptive survey using an anonymous questionnaire distributed to general practitioners and advanced practice nurses working in outpatient settings. Participants were asked to use the ZFS during routine renewal consultations in patients aged 65 years and older and to compare it with the Clinical Frailty Scale (CFS). Perceived feasibility, relevance, ease of use, reproducibility, and time required for completion were assessed. Fourteen practitioners provided usable responses, evaluating 126 older patients. The majority of participants reported that the ZFS could be completed in less than 5 minutes and considered it easy to use and reproducible. Most practitioners perceived the ZFS as relevant for routine clinical practice and feasible based on information available in medical records. A large proportion of respondents indicated their intention to integrate the ZFS into daily practice. Overall perceptions of feasibility and ease of use were comparable between the ZFS and the CFS. This study suggests that the Zulfiqar Frailty Scale is a feasible and well-accepted tool for frailty screening in primary care settings. Its simplicity and short administration time may facilitate the integration of frailty screening into routine outpatient practice. Larger studies are warranted to further assess its implementation and impact across diverse primary care contexts.

## INTRODUCTION

Older adults represent the most clinically heterogeneous age group. While many remain in good health into old age, a significant proportion are frail [1]. Frailty in older adults is a major public health issue, particularly due to the increased use of healthcare services. It is characterized by a progressive decline in functional capacity, resulting in limitations in activities of daily living [2]. This syndrome results from the complex interaction of multiple factors, including psychosocial and socioeconomic status, isolation, malnutrition, lack of appropriate physical activity, multimorbidity, and the presence of chronic diseases. These factors contribute to the onset of vulnerability, resulting from the progressive, cumulative decline of multiple physiological systems over a lifetime. It is essential to assess this syndrome in older people to intervene early in its management and prevent its main complications (falls, loss of independence, hospitalization, institutionalization, mortality), thereby improving their quality of life and reducing healthcare costs [3].

At present, there is no uniformly validated and agreed-upon screening tool. The lack of a standardized and valid method for screening frail individuals, which would enable effective targeting of care, is a major obstacle to the prevention and management of frailty in older adults.

Furthermore, a clear definition of frailty is essential for the effective care of older people. However, no formal consensus has yet been reached [4]. This difficulty stems in particular from the fact that frailty cannot be considered either a strictly normal consequence of aging or a pathology in its own right, given its variable clinical manifestations.

Over the past few decades, numerous definitions have been proposed, and several screening tools have been developed to better understand this complex syndrome. However, this diversity reflects the persistent lack of consensus on the operational definition of frailty and how to identify it in clinical practice [4]. In general practice, this issue is all the more crucial, as screening must be integrated into limited consultation time using simple, rapid, and easily reproducible tools.

In this context, the Zulfiqar Frailty Scale (ZFS) was developed specifically for outpatient practice, aiming to address the constraints of general practice while maintaining a multidimensional approach to frailty. However, beyond its psychometric performance, the implementation of a screening tool also depends on its acceptability, perceived feasibility, and suitability for professional practice.

This study is therefore part of a field evaluation process aimed at analyzing the perception of general practitioners and advanced practice nurses regarding the relevance and feasibility of implementing the ZFS in outpatient care, as well as its concordance with a commonly used reference scale, the Clinical Frailty Scale (CFS).

## MATERIAL AND METHODS

### Study objective

The main objective of this study was to evaluate perceptions of the relevance and feasibility of implementing the new ZFS frailty screening scale, which is reliable, quick, and easy to use in outpatient settings by general practitioners and advanced practice nurses (APNs). The secondary objective of the study was to analyze concordance with the CFS scale, one of the scales that is easy to use in outpatient care.

The aim is to raise awareness of frailty syndrome screening and improve access through the use of a tool adapted to outpatient medicine in order to predict the occurrence of events (such as a fall, a change in treatment, weight loss, or social change), which would allow for early intervention in the loss of autonomy and *ultimately* lead to the development of screening and management consultations for frailty syndrome in outpatient medicine.

### Study design

The study was a cross-sectional descriptive survey conducted using a completely anonymous questionnaire (Supplementary File 1) via Google Forms. An Excel file was also made available to facilitate data collection. The questionnaire was distributed to general practitioners in private practice and to advanced practice nurses in multidisciplinary health centers. Participants were contacted by telephone and email.

Supplementary File 1

We proposed testing the ZFS scale. Patients had to be assessed during a renewal consultation and be over 65 years of age. We then compared it with the CFS scale, using a sample of 5 to 10 patients per examiner. The two scales evaluated (Rockwood’s CFS scale and the ZFS scale) had to be administered during the same renewal consultation with these patients.

### Presentation of the Zulfiqar scale (ZFS)

**Figure 1:**
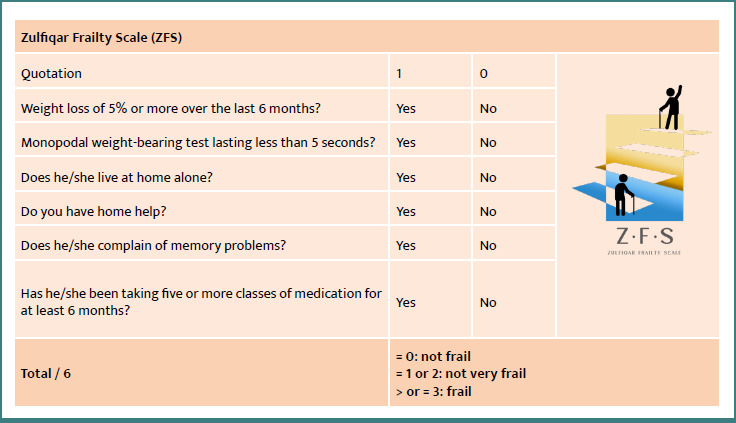
ZFS scale

The Zulfiqar Frailty Scale (Figure 1) was developed by Dr. Zulfiqar Abrar-Ahmad to serve as a screening tool for frailty syndrome, adapted for ambulatory medicine.

This consists of six items, each identified in the literature as significantly and independently associated with a poor prognosis in terms of morbidity and mortality risk [5-7]. They were also chosen for their simplicity and speed of implementation, since none of them requires prior training or specific equipment.

Each positive response (“yes”) is scored 1, and each negative response (“no”) is scored 0, yielding a total score of 0 to 6. The patient is considered “non-fragile” if the score is 0, “not very fragile” if the score is 1 or 2, and “fragile” if the score is 3 or higher. In our study, the “slightly fragile” group is merged with the “non-fragile” group.

The ZFS scale has been the subject of several studies to ensure its validity and reproducibility [8-13].

### Presentation of the CFS scale

The Clinical Frailty Scale (CFS) predicts mortality risk in the elderly based on frailty. It is used in Anglo-Saxon hospitals for in-patient care, particularly in intensive care units [14,15], but also during the Covid-19 crisis in a triage and orientation role [16-18]. More marginally, it is also used in cardiology [19,20], orthopedics [21], geriatrics [22,23], and other departments [24]. Its use in general medicine is not reported in the literature.

There are nine stages, ranging from 1 for a person in good health to 9 for a patient in the “terminal phase,” i.e., with a life expectancy of less than 6 months. The stages are defined by the presence of various clinical criteria: the patient’s general condition, the use of technical aids to move around, dependence on external human assistance (housework, carrying meals, help with dressing, grooming, organizing treatment intake), and comorbidities. The patient is considered “non-fragile” from stage 1 to stage 3, and “fragile” from stage 4 to stage 9 [25]. The stages are: 1–Very Fit; 2–Well; 3–Managing Well; 4–Living with Very Mild Frailty; 5–Living with Mild Frailty; 6–Living with Moderate Frailty; 7–Living with Severe Frailty; 8–Living with Very Severe Frailty; 9–Terminally Ill. For scores of 5 or more, the elderly patient was considered by CFS to be “frail.”

The study was registered with the French National Commission for Information Technology and Civil Liberties (CNIL; Registration No.: 2227749) and approved by the Ile de France VI Ethics Committee (registration number: 2022-A03779-22).

### Questionnaire outline and data collected

First, we collected characteristics of the sample population studied:

- Professional status (general practitioners or advanced practice nurses).
- Gender.
- Age.
- Whether or not they used frailty screening scales in their practice. If so, which ones and in what situations.

We then introduced the two scales to be administered to people aged 65 or older during a renewal consultation. Demographic data were collected in advance, including the patient’s age and gender.

We then asked them to evaluate the time required to complete the assessment, as well as the relevance, reproducibility, and ease of use of the ZFS scale compared with the CFS scale.

Finally, we specifically asked participants whether the ZFS scale was feasible based on data from patients’ medical records and whether they would consider applying it in their daily practice.

## RESULTS

We collected 16 responses to our questionnaire. Of these responses, 14 were usable.

### Characteristics of the population and patients

- Characteristics of participants (results summarized in Table 1):
  Regarding professional status, 15 participants were general practitioners (93.8%) and only one was an advanced practice nurse (6.2%). The gender ratio was 1:1.
  Participants’ ages ranged from 28 to 55, with the majority in the 30-39 age group (38%). The other age groups were distributed as follows: 6% for 20–29-year-olds, 31% for 40-49-year-olds, and 25% for 50-59-year-olds.
  Regarding the use of frailty scales, three participants (18.8%) incorporated a screening tool into their clinical routine, compared with 13 non-users (81.3%; Figure 3). The situations that led them to use it primarily involved falls among elderly patients.
- Characteristics of the patients studied (results summarized in Table 2):
  A total of 126 patients were included in the study, comprising 56 men (44.4%) and 70 women (55.6%). In terms of age distribution, 51 patients (40.5%) were between 65 and 75 years old, 54 patients (42.9%) were between 76 and 85 years old, and 21 patients (16.7%) were 86 years old or older.

**Table 1:** Summary of participant characteristics

	Total population n = 16
**Age** Between 20 and 29 Between 30 and 39 Between 40 and 49 Between 50 and 59 60 and over	1 (6.25%) 6 (37.50%) 5 (31.25%) 4 (25%) 0 (0.00%)
**Gender** Male Female	8 (50%) 8 (50%)
**Professional status** General practitioner Advanced practice nurse	15 (93.75%) 1 (6.25%)
**Use of screening scale** Yes No	3 (18.75%) 13 (81.25%)

**Figure 2:**
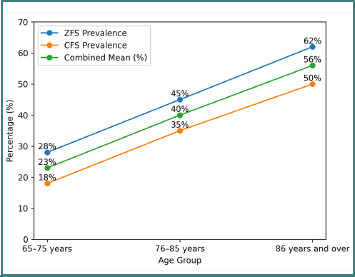
Prevalence of frailty

**Figure 3:**
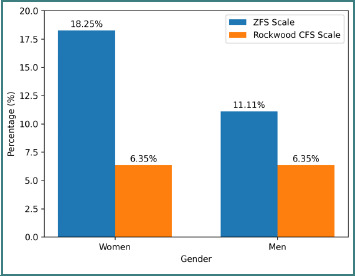
Prevalence of frailty according to patient gender

**Table 2:** Summary of patient characteristics

	Total patients n = 126
**Age** Between 65 and 75 Between 76 and 85 86 years and older	51 (40.48%) 54 (42.86%) 21 (16.67%)
**Gender** Male Female	56 (44.4%) 70 (55.6%)

### ZFS scale test

Fourteen examiners tested the ZFS scale on 126 patients.

Regarding the scale rating, 25 patients (19.8%) did not meet any frailty criteria, 64 patients (50.8%) met one or two criteria, 19 patients (15.1%) met three criteria, 13 patients (10.3%) met four criteria, four patients (3.2%) had five criteria, and one patient (0.8%) had all criteria.

Based on these data, patients were classified into three groups according to their degree of frailty, with a “pre-frailty” threshold defined as a score of 1 or 2 and a “frailty” threshold defined as a score of 3 or higher. Among the patients evaluated, 25 (19.8%) were considered “non-frail,” 64 patients (50.8%) were identified as “pre-frail,” and 37 patients (29.4%) were identified as “frail” (Table 3).

**Table 3:** Data collection using the ZFS scale

	Total patients n = 126
**ZFS score** 0 1 2 3 4 5 6	25 (19.8%) 32 (25.4%) 32 (25.4%) 19 (15.1%) 13 (10.3%) 4 (3.2%) 1 (0.8%)
**Classification of frailty** Non-frail Pre-frail Frail	25 (19.8%) 64 (50.8%) 37 (29.4%)

We then assessed the examiners’ perceptions of the ZFS scale (results summarized in Table 4).

**Table 4:** Participants' evaluation of the ZFS tool

	Total population n = 14
**Time to complete** Less than 5 minutes Between 5 and 10 minutes More than 10 minutes	8 (57%) 6 (43%) 0 (0.00%)
**Relevance** Not very relevant Very relevant	2 (14%) 12 (86%)
**Reproducibility** Reproducible Not reproducible	14 (100%) 0 (0.00%)
**Simplicity of the tool** Easy to use Difficult to use	14 (100%) 0 (0.00%)
**Feasibility based on data extracted from patient medical records** Yes No	13 (93%) 1 (7%)
**Intention to apply the ZFS scale in practice** Yes No	13 (93%) 1 (7%)

The time required to complete the test was less than 5 minutes for eight cases (57% of cases) and between 5 and 10 minutes for six cases (43% of cases).

Regarding the perceived relevance of the tool, 12 evaluators (86%) rated it as “very relevant,” while 2 (14%) did not share this opinion and found it to be of little relevance.

All participants, 14 in total, attested to its ease of use and reproducibility.

In addition, 13 participants (93%) felt that the tool could be implemented using data extracted from patients’ medical records and plan to incorporate this scale into their routine clinical practice.

### Comparison with Rockwood’s CFS scale

We then asked the examiners to complete the CFS scale.

Patients were classified according to their overall health status using the CFS tool: eight patients (6.35%) were considered to be in “very good health,” 25 patients (19.84%) were considered “healthy,” 54 of them (42.86%) were considered “fairly well,” 23 patients (18.25%) were considered “vulnerable,” seven patients (5.56%) were considered “slightly frail,” five patients (3.97%) were “moderately frail,” four patients (3.17%) were “severely frail,” and none were classified as “very severely frail” or “terminally ill.”

The frailty threshold for the CFS scale is a score of 5 or higher. Based on these data, 110 patients (87.30%) were classified as “non-frail,” while 16 patients (12.70%) were considered “frail” (Table 5).

**Table 5:** Data collection using Rockwood's CFS scale

	Total patients n = 126
**Rockwood CFS score** 1 2 3 4 5 6 7 8 9	8 (6.35%) 25 (19.84%) 54 (42.86%) 23 (18.25%) 7 (5.56%) 5 (3.97%) 4 (3.17%) 0 (0.00%) 0 (0.00%)
**Classification of frailty** Non-frail Frail	110 (87.30%) 16 (12.70%)

We then evaluated perceptions of Rockwood’s CFS scale relative to the ZFS scale (Table 6).

**Table 6:** Comparison of perceptions between the ZFS and CFS scales

	ZFS	CFS
**Time to complete** Less than 5 minutes Between 5 and 10 minutes More than 10 minutes	8 (57%) 6 (43%) 0 (0.0%)	9 (64%) 5 (36%) 0 (0.0%)
**Relevance** Not very relevant Very relevant	2 (14%) 12 (86%)	6 (43%) 8 (57%)
**Reproducibility** Reproducible Not reproducible	14 (100%) 0 (0.0%)	13 (93%) 1 (7.0%)
**Simplicity of the tool** Easy to use Difficult to use	14 (100%) 0 (0.0%)	13 (93%) 1 (7.0%)

Regarding the time required to complete the CFS tool, nine participants (64%) considered it feasible in less than 5 minutes, and five others (36%) estimated that it would take between 5 and 10 minutes.

Regarding the assessment of the CFS’s relevance, six examiners (43%) considered it to be of little relevance, while eight (57%) considered it to be very relevant.

The reproducibility of the score in the office was rated positively by 13 participants (93%), compared to only one participant (7%) who rated it non-reproducible.

Finally, 13 evaluators (93%) found the scale easy to use, while only 1 (7%) found it difficult.

The prevalence of frailty, defined according to the thresholds of each scale, varied by age group (Figure 2). For patients aged 65 to 75, it was estimated at 29% using the ZFS and 18% using the CFS. Among those aged 76 to 85, the prevalence was 46% for the ZFS and 35% for the CFS. Finally, among patients aged 86 and over, frailty affected 62% according to the ZFS and 50% according to the CFS (Table 7).

**Table 7:** Comparison of the prevalence of frailty between the ZFS and CFS scales

Age group	ZFS prevalence	CFS prevalence
65–75	29	18
76–85	46	35
86 years and older	62	50

Among the 126 patients, 23 women (18.25%) were considered “frail” according to the ZFS score, compared with eight women (6.35%) according to the CFS score. Among men, the prevalence was 11.11% (14 patients) for the ZFS scale and 6.35% (eight patients) for the CFS (Figure 3).

## DISCUSSION

In many countries, general practitioners see their patients in their own environment, which allows them to assess the interaction between their patients’ clinical and environmental conditions. Their preventive role is based on constant vigilance, personalized patient advice, and regular follow-up to anticipate risks and promote independence. In this sense, they are at the heart of a proactive approach aimed at preserving overall health and preventing the worsening of frailty.

Regarding the study results, we first highlight a difference in participants’ ages between those who used frailty screening scales and those who did not. The majority of clinicians who used screening scales were in the first two age groups (20-29 and 30-39). Therefore, it would be necessary to expand the study to include a larger number of participants to determine whether younger practitioners are more likely to use these scales in their daily practice. We also noted a contrast between the proportion of participants currently using frailty scales (18.8% of respondents) in the preliminary questions and the proportion considering incorporating the ZFS scale into their future practice (93% answered “yes”) at the end of the questionnaire. This could reflect a lack of awareness of this syndrome and its screening among practitioners, or a lack of practical tools available to them. Few studies have evaluated the impact of frailty screening on patient care, which may explain the lack of interest and knowledge of this concept among general practitioners. Further research could increase the visibility of this approach. Furthermore, our study could be extended to evaluate the integration of the ZFS screening tool among other healthcare professionals (nurses, pharmacists, social workers) in order to raise their awareness of preventive measures.

Secondly, based on our data, we find that the prevalence of frailty syndrome increases with patient age. In addition, the prevalence of frailty is higher in women, according to the ZFS scale (18.25% compared to 11.11% in men). These results are consistent with current literature on the prevalence of frailty [11].

Finally, comparisons between the two scales show similar results in terms of completion time, reproducibility, and ease of use. The main difference concerns the perception of relevance, with the ZFS scale being considered more relevant (86% vs. 57% for the CFS).

We can note the strengths of the ZFS tool. It has been the subject of initial studies showing good correlation with reference scales, such as the Fried scale [11], the CFS scale [12], and the mSEGA score [13]. A significant positive correlation (r = 0.73; *P* < 0.0001) between ZFS and mSEGA scores confirms the validity of the ZFS tool, consistent with the literature and highlighting the importance of comprehensive, multidimensional frailty assessment tools [13]. These two scales effectively assess the physical, cognitive, and social dimensions of frailty, reinforcing their usefulness in clinical practice. The significant impact of falls and hospitalizations on frailty classification by the ZFS and mSEGA scales is an essential element, as these factors are widely recognized as major predictors of frailty and adverse clinical complications in older adults. The ZFS tool has high sensitivity and negative predictive value, significantly reducing the risk of false negatives and limiting unnecessary referrals to specialist consultations [8]. These results attest to the reliability and effectiveness of the ZFS for screening for frailty in general practice.

It is also notable for its speed of administration, with an average completion time of approximately 72 seconds [11], demonstrating its feasibility in routine practice. In our study, 57% of participants estimated that it took less than 5 minutes to complete the scale. Therefore, our results are consistent with previous studies.

In addition, the questions are simple, based on information accessible to any healthcare professional, and the tool requires no prior training, specific equipment, or special facilities, making it a pragmatic screening tool that can be integrated into general practice.

Several limitations should be acknowledged. First, the number of participating professionals was small, which limits the generalizability of the findings. Participation was voluntary, potentially introducing selection bias, as clinicians with an interest in geriatrics or frailty may have been more inclined to respond. Second, although a substantial number of patients were assessed, evaluations were clustered by examiner, which may have influenced the results. Third, some items of the ZFS rely on patient-reported information, particularly regarding cognitive complaints, which may be subject to reporting bias or optimism bias. Finally, this study did not assess clinical outcomes; therefore, no conclusions can be drawn regarding the scale’s predictive value.

Despite these limitations, the strength of this study lies in its pragmatic design and its focus on real-world implementation. The ZFS does not aim to replace a comprehensive geriatric assessment, but rather to serve as an initial screening tool to identify patients who may benefit from further evaluation. Its simplicity and feasibility may facilitate broader adoption of frailty screening in primary care, including by non-physician healthcare professionals.

Future research should involve larger and more diverse samples of primary care practitioners and explore the integration of the ZFS into routine workflows. Prospective studies assessing its impact on clinical decision-making and patient outcomes would also be valuable.

## CONCLUSION

Screening for frailty syndrome can seem complex to general practitioners, who need simple, practical tools. These tools must be easily integrated into daily practice to enable rapid, reliable detection of frailty, thereby enabling referral of at-risk patients for more in-depth assessments and the development of appropriate care. Our study highlights a lack of awareness of frailty syndrome among practitioners. It therefore seems essential to reinforce this teaching in the initial training of future general practitioners to develop a reflex for early prevention in elderly patients consulting in outpatient settings and to improve knowledge of appropriate care modalities.

The ZFS scale, designed specifically for general practice, is non-intrusive, quick to administer, and focused on clinical observation, which perfectly meets the requirements and constraints of routine consultations. Its simplicity and feasibility make it a particularly suitable tool for assessing frailty in everyday practice. The implementation of this scale in general practice offers a promising prospect for improving the early detection of frailty.

Given the existence of medical deserts, screening must also rely on paramedical professionals using simple, standardized tools. The ZFS tool can be routinely integrated by general practitioners, but could also be administered by nurses, physical therapists, occupational therapists, or social workers. Pharmacists should also play a role in promoting the health of frail individuals, particularly through screening and long-term follow-up interventions. Our study could therefore be extended to various healthcare professionals in order to evaluate its implementation and feasibility in the paramedical sector.

In this era of digital development, and following the example of the ICOPE project, it seems essential to develop connected tools that are accessible everywhere and to everyone, with a view to creating a preventive care model.

## Data Availability

The datasets used and/or analyzed during the current study are available from the corresponding author upon request.
