# Supplementary material for: Acceptability and feasibility of the Zulfiqar Frailty Scale (ZFS) in primary care: a cross-sectional survey
Source: J Med Life. 2026 Mar;19(3):187–93. doi: 10.25122/jml-2025-0175 (PMC13155165; doi:10.25122/jml-2025-0175)
Supplement: Supplementary file 1 [file JMedLife-19-187-s001.pdf]

QUESTIONNAIRE ON THE IMPLEMENTATION OF THE ZULFIQAR FRAILTY SCALE (ZFS) IN GENERAL PRACTICE

**Professional status** (general practitioner or advanced practice nurse)
**Gender of professional** (Male/Female/Prefer not to answer)
**Age of professional**
**Do you use frailty screening scales?**
**If so, which ones and in what situations?**

**FRAILTY ASSESSMENT**

**Patient age**
**Patient's gender** (Male/Female)
**ZFS SCORE ranging from 0 to 6**
**1. Weight loss of ≥ 5% of usual weight in 6 months:** YES/NO
**2. Pathological single-leg stance test (both feet without shoes) < 5 seconds:** YES/NO
**3. Taking ≥ 5 therapeutic classes (in the case of drugs combining several molecules, count each molecule as 1 therapeutic class):** YES/NO
**4. Does he/she live alone at home:** YES/NO
**5. Presence of home help (including family help):** YES/NO
**6. Does he/she complain of memory problems (answer may be given by a caregiver):** YES/NO
**Total out of 6**
**ROCKWOOD CFS SCORE rated 1 to 9**
**Score out of 9**

**COMPARISON OF ZFS SCALE VS. ROCKWOOD CFS SCALE**

**Time taken to complete the score** (< 5 min / 5 to 10 minutes / > 10 minutes)
**Relevance of the score** (Not relevant / Not very relevant / Very relevant)
**Reproducibility of the score in the office** (Not reproducible / Reproducible)
**Ease of use of the score** (Difficult / Easy)

**About the ZFS scale:**
**Is it feasible to use data from patient medical records?**
**Do you plan to apply the ZFS scale in your practice?**

© 2026 by the authors. This article is an open access article distributed under the terms and conditions of the Creative Commons Attribution (CC BY 4.0) license.
